# Malignant Transformation of a Hormone Receptor-Positive Benign Intracranial Meningioma During Pregnancy: A Case Report

**DOI:** 10.7759/cureus.97564

**Published:** 2025-11-23

**Authors:** Walter Fagundes, Oguz Kagan Sahin, Rohan Sabloak, Yasmin P Silva, Fernando Luiz R Dantas

**Affiliations:** 1 Neurosurgery, Geneuro - International Research Group in Neuroscience, Vitória, BRA; 2 Neurosurgery, Federal University of Espírito Santo, Vitória, BRA; 3 Neurosurgery, Geneuro - International Research Group in Neuroscience, Istanbul, TUR; 4 Neurosurgery, Geneuro - International Research Group in Neuroscience, Palembang, IDN; 5 Neurosurgery, Geneuro - International Research Group in Neuroscience, Selfoss, ISL; 6 Neurosurgery, Geneuro - International Research Group in Neuroscience, Belo Horizonte, BRA

**Keywords:** malignant, meningioma, pregnancy, progesterone receptor, recurrence

## Abstract

Meningiomas are predominantly benign, more prevalent in women, and related to sex hormones. Malignant transformation during pregnancy, however, is exceptionally rare. We report the case of a 22-year-old woman with a WHO Grade I fibrous meningioma that was completely resected. One year later, during pregnancy following ovulation induction therapy, she developed a recurrence, histologically confirmed as a WHO Grade II atypical meningioma. After delivery, a second recurrence occurred, with progression to WHO Grade III anaplastic meningioma. Immunohistochemistry consistently revealed progesterone receptor (PR) positivity and absence of estrogen receptor (ER) expression. This case represents one of the rare documented instances of malignant transformation of a benign meningioma during pregnancy without prior radiotherapy. It emphasizes the potential influence of pregnancy-related hormonal changes in tumor progression and highlights the importance of close surveillance with multidisciplinary management in reproductive-age women with meningiomas.

## Introduction

Meningiomas account for approximately 25% of all primary intracranial neoplasms [1]. Although meningiomas are usually benign tumors, they have the potential for progression and aggressive behavior. Recurrence is not limited to those with atypical or malignant histological features [2]. Benign meningiomas (Grade I) are about twice as common in women, suggesting a hormonal influence in their development and progression, although atypical (Grade II) and anaplastic (Grade III) meningiomas are predominant in men [3].

Molecular biology has revealed the expression of hormone receptors in meningiomas, particularly progesterone receptors (PRs), although estrogen and growth hormone receptors have also been detected [4]. PRs have been observed in normal arachnoid tissue; however, normal adult meninges express low levels of PRs [2]. However, the role of receptor status in tumor progression and malignant transformation remains unclear [3]. Previously described cases of tumor expansion, malignancy, and/or recurrence after resection during pregnancy suggest that exposure to high levels of progesterone may be involved in the physiopathology of these tumors [5]. Furthermore, it has been shown that pregnant patients and those exposed to exogenous hormone replacement therapy present rapid growth of meningiomas, suggesting that sex steroids play an important role in tumor growth [6]. Although tumor enlargement during pregnancy has been reported, cases of rapid recurrence with histological progression from benign to malignant meningioma are exceedingly rare.

Here, we report a case of intracranial meningioma recurrence during pregnancy, followed by malignancy. This case raises the clinical question of whether pregnancy-related hormonal changes can drive not only recurrence but also malignant transformation of a benign meningioma. To our knowledge, such progression without prior radiotherapy is exceptionally rare, emphasizing the importance of hormone receptor evaluation and close multidisciplinary monitoring in reproductive-age women with meningiomas. We also describe the clinical and pathophysiological aspects of this case.

## Case presentation

**Figure 1:**
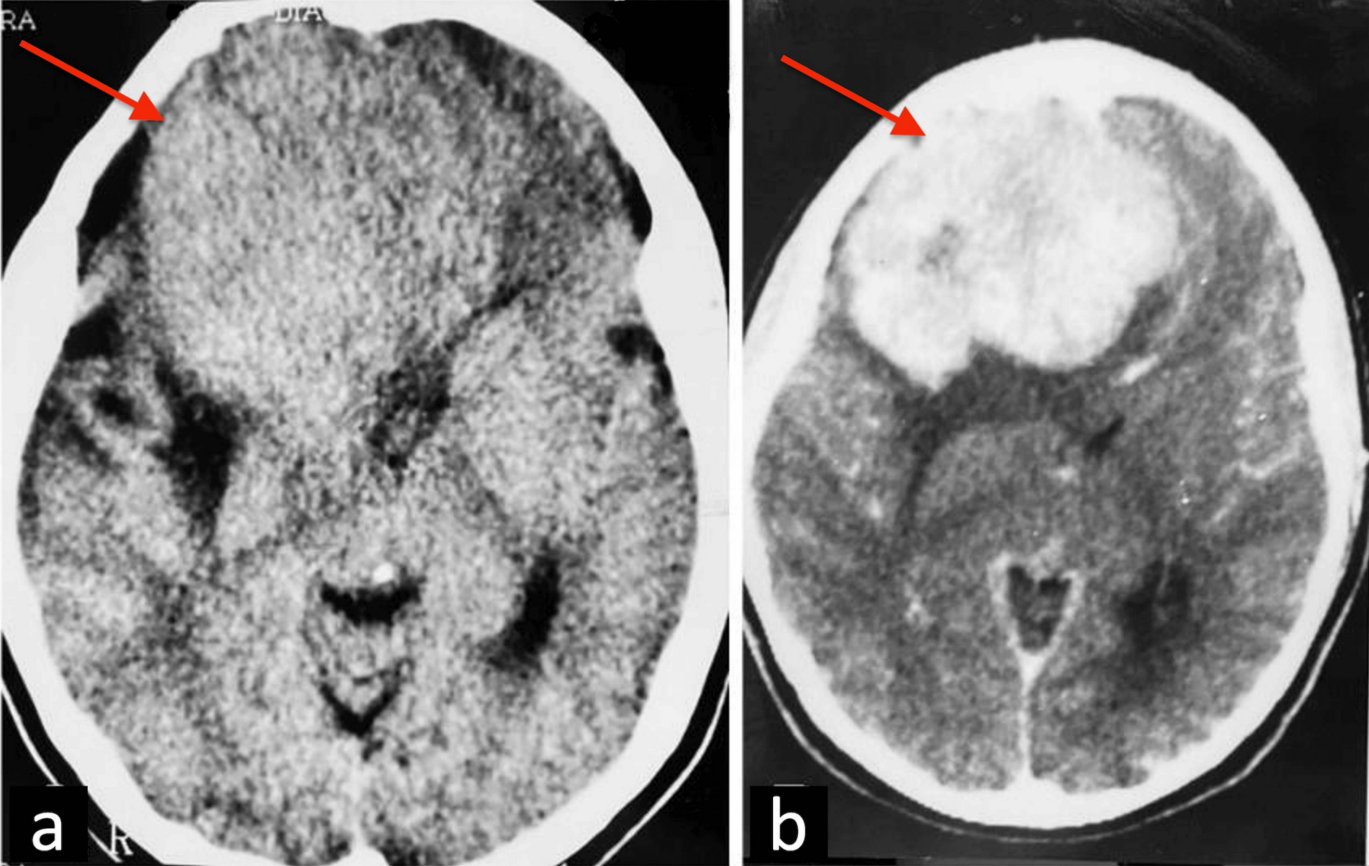
Brain CT findings a: brain CT scan revealed a large right frontal tumor (red arrow); b: with homogeneous contrast enhancement (red arrow).

A 22-year-old woman presenting with bilateral visual impairment and right eye anisocoria was admitted to the Neurosurgery Department of Santa Casa of Belo Horizonte, Brazil. A brain computed tomography (CT) scan revealed a large isodense tumor with homogeneous contrast enhancement in the right frontal region (Figures 1a, 1b).

**Figure 2:**
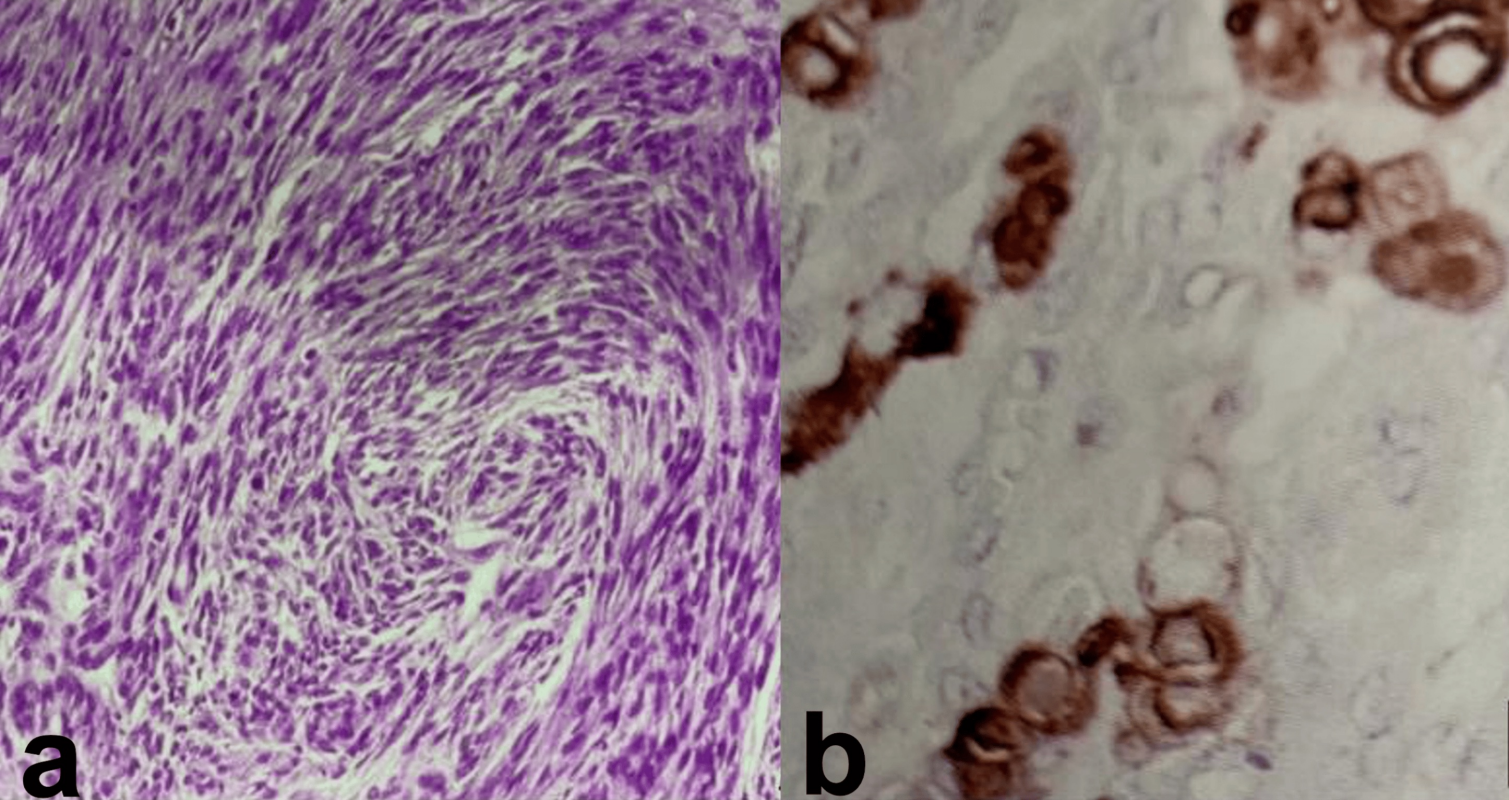
Histopathological and immunohistochemistry findings a: histopathological study revealed a fibrous meningioma; b: immunoreactivity for epithelial membrane antigen (EMA).

She underwent total surgical resection, and histopathology confirmed a fibrous meningioma (WHO Grade I) (Figure 2a). The histological grade of the tumor was determined according to the WHO 2021 classification. Immunohistochemistry showed marked reactivity for epithelial membrane antigen (Figure 2b) and vimentin positivity.

**Figure 3:**
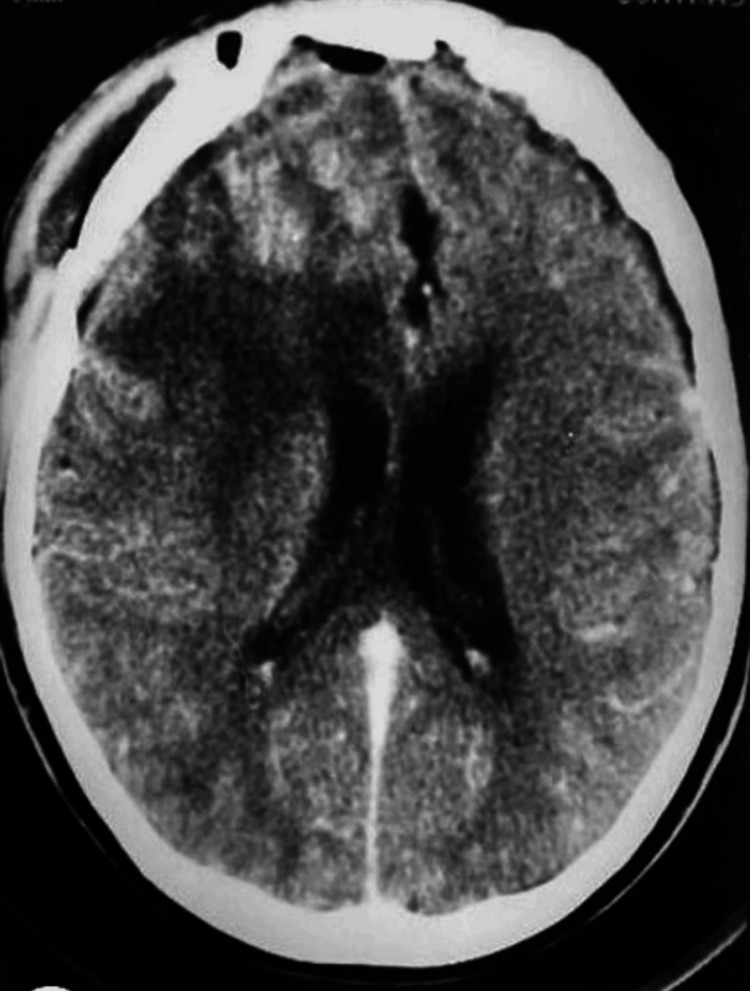
Postoperative brain CT scan after nine months showed no tumor

A follow-up brain CT scan performed nine months later showed no recurrence (Figure 3).

**Figure 4:**
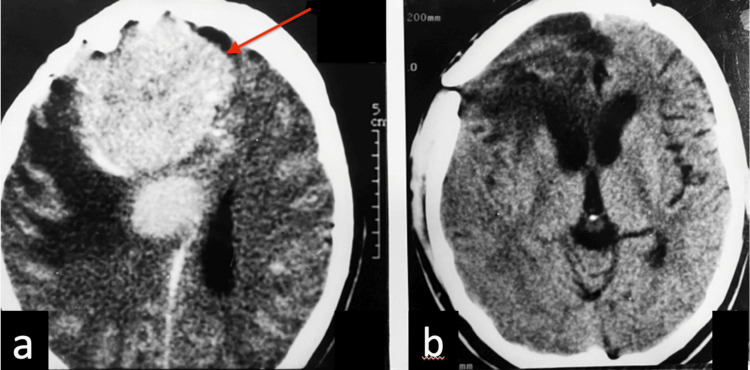
Brain CT findings a: brain CT scan showed tumor recurrence (red arrow) in the right frontal region and a new right parietal lesion; b: postoperative brain CT scan showed no tumor.

One year after the surgery, the patient, who was pregnant at that time, was following hormone replacement therapy and ovulation induction with clomiphene, presented with headaches associated with nausea, vomiting, and fatigue. A repeat CT scan revealed tumor recurrence in the right frontal and parietal parasagittal regions (Figure 4a), which were surgically treated. Histological examination confirmed atypical meningioma (WHO grade II), and a subsequent CT scan confirmed complete excision of the lesions (Figure 4b). In the postoperative period, the patient developed left hemiparesis, characterized by reduced motor strength.

**Figure 5:**
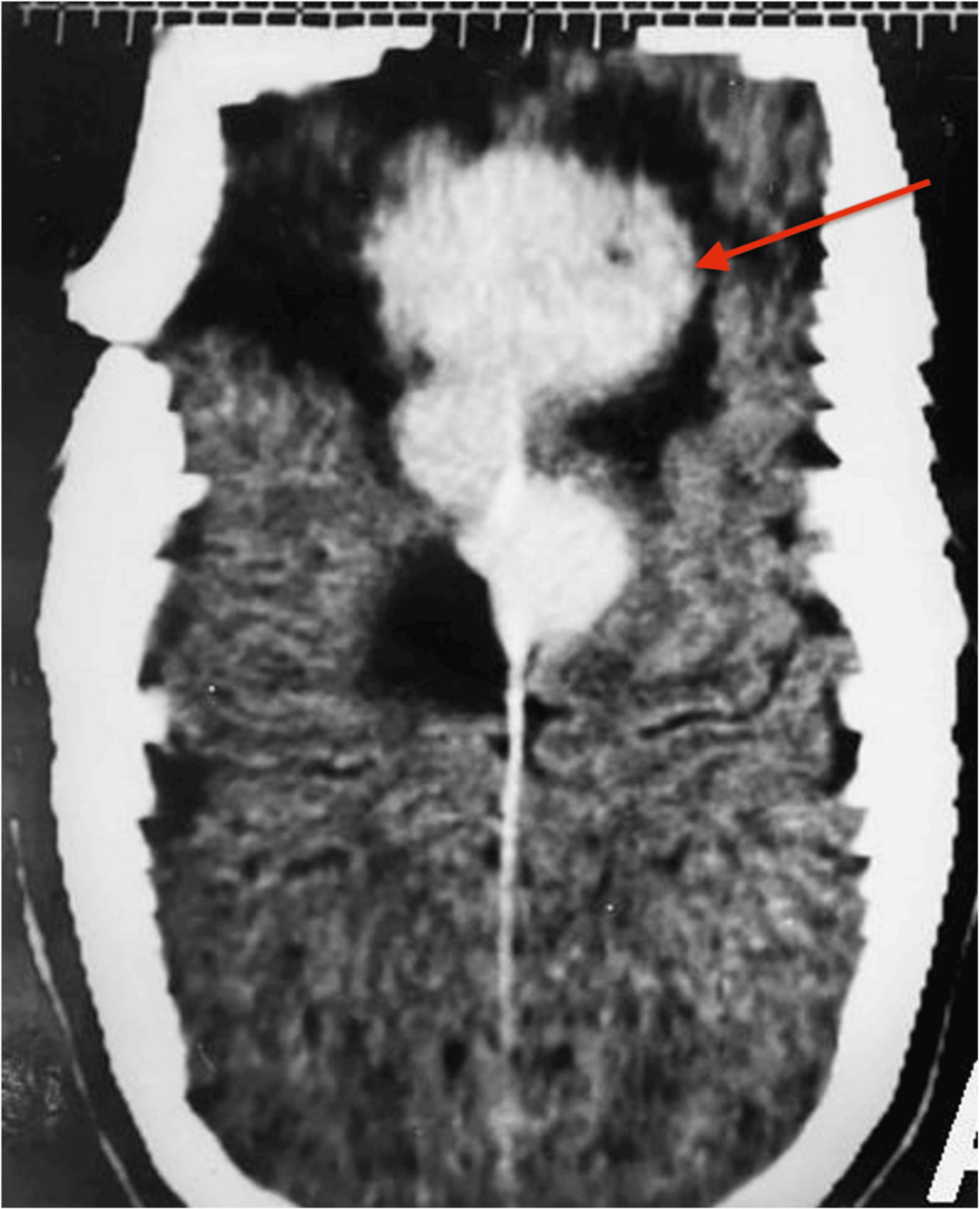
In the puerperium, the brain CT scan showed a recurrence of the tumor (red arrow)

**Figure 6:**
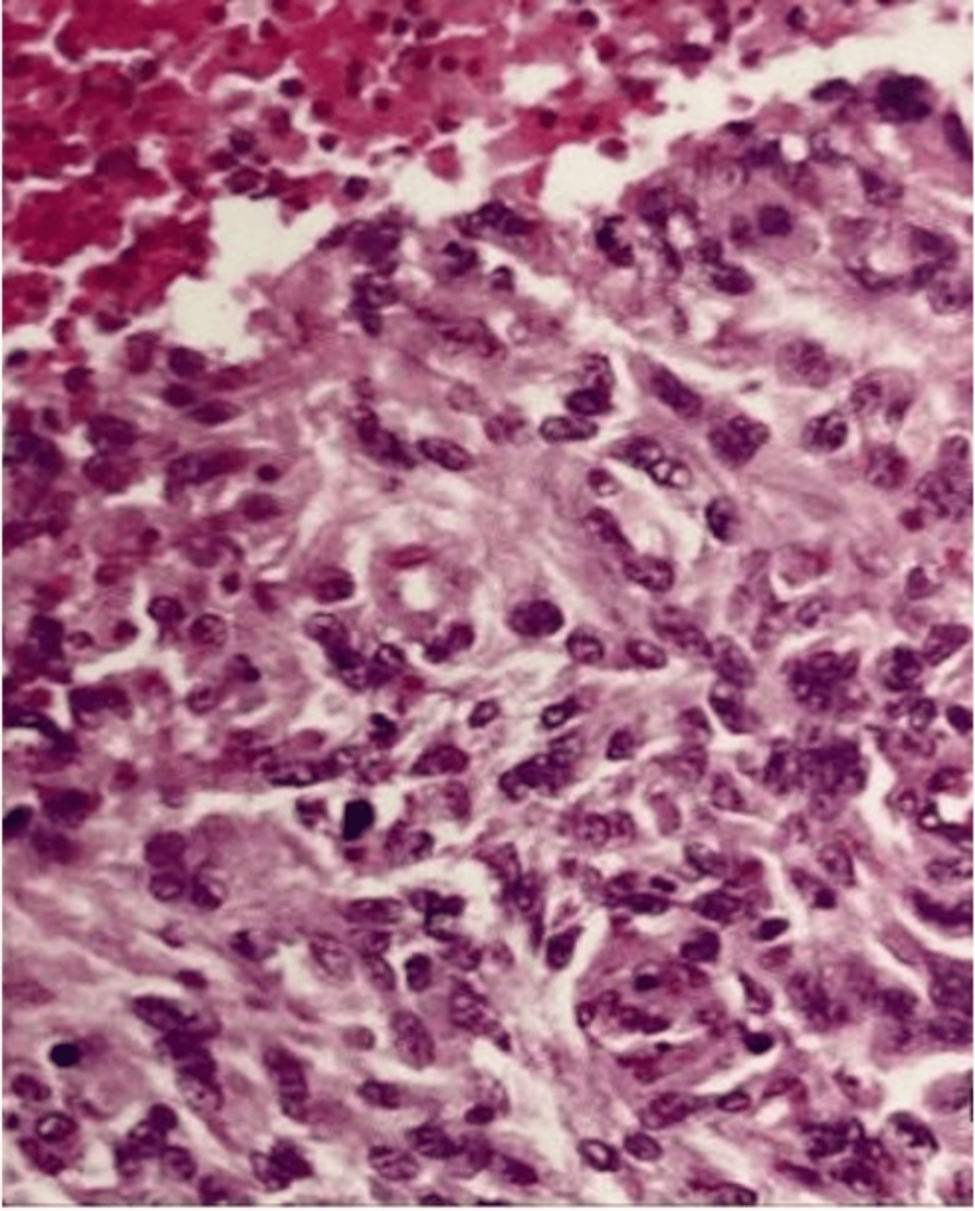
Histopathological study showed an anaplastic meningioma with nuclear polymorphism, numerous mitoses, and necrosis

At 32 weeks of gestation, pregnancy was interrupted due to eclampsia. In the puerperium, CT again demonstrated recurrence of the right frontal and parasagittal parietal lesions (Figure 5), which were once again surgically completely resected. Histological analysis revealed anaplastic (malignant) meningioma progression (WHO Grade III) (Figure 6). One month after the third surgery, a follow-up CT scan showed the presence of a new tumor, now in the left parietal region, which led to the initiation of radiotherapy. After eight months, the patient was admitted to the hospital with somnolence and paraparesis. Brain magnetic resonance imaging showed an increase in the previous left parietal lesion. She underwent complete tumor resection using a new surgical approach. The patient displayed an improvement in her level of consciousness in the postoperative period, albeit maintaining motor deficits.

**Figure 7:**
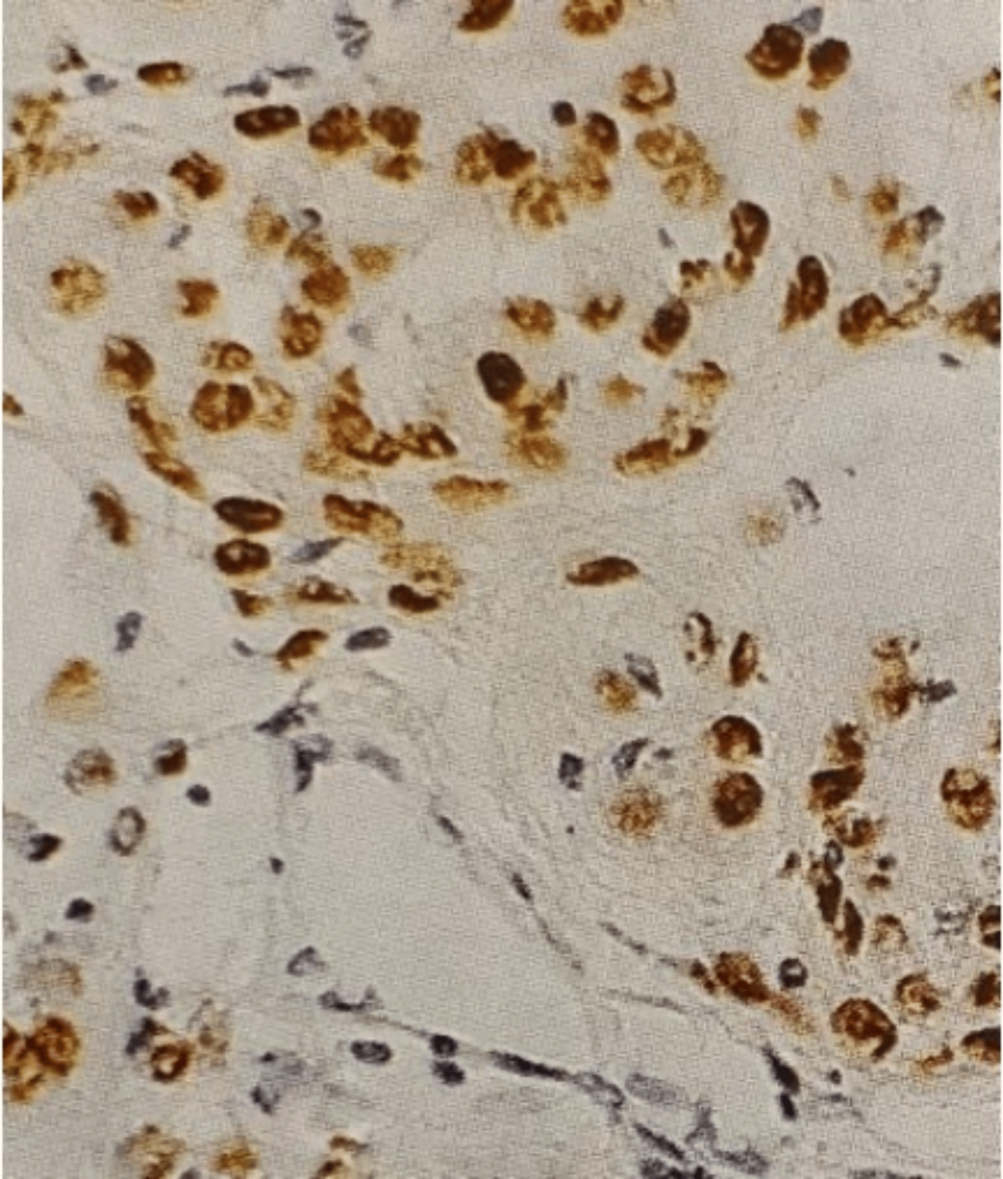
Immunohistochemical staining Immunohistochemical staining of a meningioma tissue sample showing strong nuclear positivity for progesterone receptors (PR) in tumor cells. The brown staining highlights PR expression, indicative of hormone receptor involvement in tumor biology. PR expression is typically higher in benign meningiomas and tends to decrease with malignant progression.

**Figure 8:**
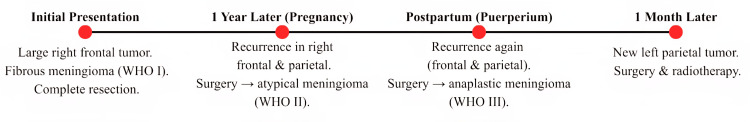
Timeline of tumor progression and interventions

After each surgery, tumor samples from different grades of meningioma were tested for the presence of ERs and PRs. The tests showed positive results for PRs (Figure 7) and negative results for ERs. The timeline of the patient’s treatment summary is also presented in Figure 8.

## Discussion

To the best of our knowledge, this is the second case report of the rapid malignant transformation of a benign meningioma during pregnancy that was not previously treated by radiotherapy. He et al. (2023) have published the first case of rapid recurrence and malignant transformation of a benign meningioma after pregnancy [5]. Our case is very similar to that of He et al., involving a young woman with a previously resected WHO Grade I meningioma that rapidly recurred and transformed malignantly following pregnancy [5]. Similar to their report, no prior radiotherapy was given, suggesting a potential role of pregnancy-related hormonal changes in driving aggressive tumor behavior. In the literature, other cases of meningioma growth or recurrence during pregnancy have been reported [7-9]. While most meningiomas express PRs and may enlarge or recur during pregnancy, cases involving rapid malignant transformation, as seen in our patient, are exceptionally rare. Unlike the more commonly described growth without histological progression, our findings expand upon the existing literature by highlighting the potential for pregnancy-associated hormonal surges to drive aggressive biological behavior in initially benign meningiomas. Therefore, our findings not only support but also build upon prior evidence by emphasizing that pregnancy-related hormonal shifts may not only drive recurrence but also contribute to aggressive histopathological progression in rare cases.

The role of ERs and PRs in meningiomas as prognostic markers, and their influence on tumor behavior, remains unclear [10-12]. Histopathological and molecular studies have shown the presence of PRs in the majority of intracranial meningiomas (84-100%) [13-15]. PR-positive is observed more frequently in benign than in malignant meningiomas [2,16]. Normal meningeal tissues do not have ERs, and ERs are uncommon in intracranial meningiomas (0-33%) [13-15].

The increase in meningioma growth rates observed in situ during pregnancy suggests a relationship between high levels of progesterone and tumor growth rate after specific hormone receptor stimulation [15,17]. Rubinstein et al. observed that among recurrent meningiomas, 92% expressed PRs and 54% expressed ERs. Notably, the mean concentration of PRs was significantly higher in the recurrent tumor group (p < 0.02) [15].

Therefore, it is important to exercise caution in patients with a history of meningioma or other hormone-dependent tumors when initiating hormonal treatment or consulting with a woman planning to become pregnant, due to the potential risks of tumor growth, recurrence, and malignant transformation [18]. Such patients should be assisted by a multidisciplinary team, considering neurosurgical, obstetric, pediatric, and endocrinological aspects [18]. Once diagnosed, patients should be closely monitored with regular clinical and imaging evaluations [4]. Surgical intervention should be considered based on the patient's clinical status, tumor location and size, gestational age, and fetal condition [19]. Adjunctive treatments, including radiotherapy and even immunotherapy, may be considered, particularly in cases of recurrence or incomplete resection and after delivery [2,13].

It is important to note that epidemiology, clinical presentation, and radiographic features do not reliably differentiate between meningioma grades [20]. While malignant tumors typically grow more rapidly, display more heterogeneous and aggressive imaging characteristics, and tend to recur earlier [13,20], the most reliable distinguishing feature is a lower apparent diffusion coefficient (ADC) value, which indicates higher cellularity [20].

Our case suggests that PR testing may have value in recurrent meningiomas, while advanced imaging tools such as ADC mapping could be of assistance in differentiating tumor grades and guiding management.

## Conclusions

This case emphasizes that meningiomas, which are hormone-dependent tumors expressing PRs, may pose significant risks of growth, recurrence, and even malignant histological transformation during pregnancy, highlighting the need for close monitoring in women with a history of meningioma who are planning to conceive. Therefore, neurosurgical, obstetric, endocrinological, and pediatric considerations must be integrated into the management of these cases. Early multidisciplinary involvement can help guide timely interventions and improve patients’ health outcomes.

## References

[1] Guevara P, Escobar-Arriaga E, Saavedra-Perez D (2010). Angiogenesis and expression of estrogen and progesterone receptors as predictive factors for recurrence of meningioma. J Neurooncol.

[2] Pravdenkova S, Al-Mefty O, Sawyer J, Husain M (2006). Progesterone and estrogen receptors: opposing prognostic indicators in meningiomas. J Neurosurg.

[3] Saitoh Y, Oku Y, Izumoto S, Go J (1989). Rapid growth of a meningioma during pregnancy: relationship with estrogen and progesterone receptors--case report. Neurol Med Chir (Tokyo).

[4] Claus EB, Calvocoressi L, Bondy ML, Wrensch M, Wiemels JL, Schildkraut JM (2012). Exogenous hormone use, reproductive factors, and risk of intracranial meningioma in females. J Neurosurg.

[5] He L, Yu S, Wang L (2023). Rapid recurrence and malignant transformation of a benign meningioma after pregnancy: a case report. Br J Neurosurg.

[6] Lee E, Grutsch J, Persky V, Glick R, Mendes J, Davis F (2006). Association of meningioma with reproductive factors. Int J Cancer.

[7] O'Higgins A, Johnson S, Kahn K, Imcha M, Slevin J (2017). A case of hormone-sensitive meningioma progressing with pregnancy. BJOG-an Int J Obstet Gynaecol.

[8] Chow MS, Mercier PA, Omahen DA, Wood SL, Johnson JA (2013). Recurrent exophytic meningioma in pregnancy. Obstet Gynecol.

[9] Shitara S, Nitta N, Fukami T, Nozaki K (2012). Tuberculum sellae meningioma causing progressive visual impairment during pregnancy. Case report. Neurol Med Chir (Tokyo).

[10] Hua L, Zhu H, Li J (2018). Prognostic value of estrogen receptor in WHO Grade III meningioma: a long-term follow-up study from a single institution. J Neurosurg.

[11] Liu F, Chen W, Chen J (2018). Letter to the Editor. Is there any relationship between estrogen receptor/progesterone receptor status and recurrence of meningioma?. J Neurosurg.

[12] Maiuri F, Mariniello G, Guadagno E, Barbato M, Corvino S, Del Basso De Caro M (2019). WHO grade, proliferation index, and progesterone receptor expression are different according to the location of meningioma. Acta Neurochir (Wien).

[13] Hilbig A, Barbosa-Coutinho LM (1998). Meningiomas and hormonal receptors. Immunohistochemical study in typical and non-typical tumors. Arq Neuropsiquiatr.

[14] Hsu DW, Efird JT, Hedley-Whyte ET (1997). Progesterone and estrogen receptors in meningiomas: prognostic considerations. J Neurosurg.

[15] Rubinstein AB, Loven D, Geier A, Reichenthal E, Gadoth N (1994). Hormone receptors in initially excised versus recurrent intracranial meningiomas. J Neurosurg.

[16] Assimakopoulou M (2000). Human meningiomas: immunohistochemical localization of progesterone receptor and heat shock protein 27 and absence of estrogen receptor and PS2. Cancer Detect Prev.

[17] Hortobágyi T, Bencze J, Murnyák B, Kouhsari MC, Bognár L, Marko-Varga G (2017). Pathophysiology of meningioma growth in pregnancy. Open Med (Wars).

[18] Benson VS, Pirie K, Green J, Bull D, Casabonne D, Reeves GK, Beral V (2010). Hormone replacement therapy and incidence of central nervous system tumours in the Million Women Study. Int J Cancer.

[19] Akeyson EW, McCutcheon IE (1996). Management of benign and aggressive intracranial meningiomas. Oncology (Williston Park).

[20] Louis DN, Scheithauer BW, Budka H (2000). World Health Organization Classification of Tumours. Pathology and Genetics of Tumours of the Nervous System. Lyon: IARC Press.

